# Polyethylene Glycol-Stabilized Zein Nanoparticles Containing Gallic Acid

**DOI:** 10.17113/ftb.60.02.22.6981

**Published:** 2022-06

**Authors:** Heliton Augusto Wiggers, Margani Taise Fin, Najeh Maissar Khalil, Rubiana Mara Mainardes

**Affiliations:** Pharmaceutical Nanotechnology Laboratory, Department of Pharmacy, Midwest State University, Alameda Élio Antonio Dalla Vecchia St, 838, 85040-167 Guarapuava, PR, Brazil

**Keywords:** zein nanoparticles, gallic acid release, polyethylene glycol, food simulants, DPPH scavenging

## Abstract

**Research background:**

Gallic acid is a polyphenol with antioxidant and antitumor activities; however, its use as a nutraceutical or drug is hindered by its low bioavailability. Zein is a natural protein found in corn and has been applied as nanoparticle drug carrier. In this study, zein nanoparticles were obtained and stabilized with polyethylene glycol (PEG) as gallic acid carriers.

**Experimental approach:**

Nanoparticles were obtained by the liquid-liquid method and characterized in terms of mean size, polydispersity index, zeta potential, morphology, solid-state interactions and encapsulation efficiency/drug loading. The stability of nanoparticles was evaluated in simulated gastrointestinal fluids and food simulants, and the antioxidant activity was determined by the scavenging of the 2,2-diphenyl-1-picrylhydrazyl (DPPH) radical.

**Results and conclusions:**

Zein nanoparticles containing gallic acid were obtained and stabilized only in the presence of PEG. Under optimal conditions, nanoparticles with mean size <200 nm, low polydispersity index (<0.25) and negative zeta potential (−20 mV) were obtained. The gallic acid encapsulation efficiency was about 40%, loading about 5%, and it was encapsulated in an amorphous state. Fourier transform infrared spectroscopy (FTIR) did not identify chemical interactions after gallic acid nanoencapsulation. Zein nanoparticles were more prone to release the gallic acid in gastric than intestinal simulated medium; however, more than 50% of drug content was protected from premature release. In food simulants, the gallic acid release from nanoparticles was prolonged and sustained. Moreover, the nanoencapsulation did not reduce the antioxidant activity of gallic acid.

**Novelty and scientific contribution:**

The results show the importance of PEG in the formation and its effect on the properties of zein nanoparticles obtained by the liquid-liquid dispersion method. This study indicates that PEG-stabilized zein nanoparticles are potential carriers for oral intake of gallic acid, preserving its antioxidant properties and enabling its use in the pharmaceutical and food industries.

## INTRODUCTION

Polyphenols are micronutrients found in plant-based foods presenting recognized antioxidant activity associated with health benefits. Studies have shown that polyphenols and their derivatives may reduce the risks of diabetes, cardiovascular and neurodegenerative diseases, besides their chemopreventive and antitumor potential (*1*, *2*). Gallic acid (3,4,5-trihydroxybenzoic acid) is a polyphenol from the class of phenolic acids found in a wide variety of foods and herbs, and its antioxidant activity is well known. Antimicrobial, anticarcinogenic and antimutagenic properties have also been described, and considerable interest has been given to its ability to protect the cells against oxidative stress (*3*-*5*). Although it has a therapeutic potential, the application of gallic acid under biological conditions is limited by its biopharmaceutical and pharmacokinetic properties, hindering its applications. Gallic acid is a polar and hydrophilic compound, which restricts its penetration through cell membranes. Thus, its low absorption associated with fast renal elimination become features that interfere with its oral bioavailability, reducing it dramatically (*6*). Considering the potential application of gallic acid in the food and pharmaceutical industries, the development of a prolonged gallic acid delivery system could improve its oral bioavailability.

Zein is a natural protein abundantly found in corn, belonging to the prolamin class. About 50% of the total amino acids present in zein are non-polar (leucine, alanine, proline and phenylalanine); however, the high glutamine content confers certain hydrophilicity, resulting in an amphiphilic protein (*7*, *8*). These characteristics allow the interaction of both hydrophobic and hydrophilic compounds with zein structure (*9*). In the last years, zein has been applied for the nanoencapsulation of drugs, nutraceuticals and other compounds (*10*-*15*). Another relevant zein feature is its resistance to digestive enzymes, resulting in slow digestibility in the gastrointestinal tract. This property has been exploited for the development of zein-controlled release systems for the oral delivery of functional compounds (*16*-*18*). However, the use of zein as a matrix for nanoparticles is not a simple issue. Due to its isoelectric point (6.2), zein aggregates at neutral pH, and thus, its stability and dispersibility are important concerns. Some attempts have been made to avoid the colloidal aggregation of zein nanoparticles, and they are based on the inclusion of a stabilizing agent (sodium caseinate is the most common or surfactants) to reduce the hydrophobic interaction between particles (*19*-*22*).

This study aims to investigate the influence of polyethylene glycol (PEG), an anionic polymer which has flexible hydrophilic chains, in the formation and stabilization of zein nanoparticles containing gallic acid, for its application in the pharmaceutical or food industry. Nanoparticles were characterized and their stability in simulated gastrointestinal fluids and in fatty food simulant was studied. Moreover, the antioxidant activity of zein-PEG nanoparticles containing gallic acid was evaluated.

## MATERIALS AND METHODS

### Materials

Zein, gallic acid (98.0% purity), polyethylene glycol (PEG; 10 kDa) and 2,2-diphenyl-1-picrylhydrazyl (DPPH) were purchased from Sigma-Aldrich Co. Ltd., Merck (St. Louis, MO, USA). Absolute ethanol was purchased from Biotec (Lages, Brazil). HPLC grade acetonitrile was purchased from LiChrosolv (Darmstadt, Germany). The solutions used in the experiments were prepared using ultrapure water (Millipore, Milford, MA, USA).

### Preparation of zein-PEG nanoparticles containing gallic acid

The nanoparticles were obtained by the liquid-liquid dispersion method (*23*), with some modifications. The influence of PEG, gallic acid, zein and hydroalcoholic solution content, and aqueous to organic phase ratio on nanoparticle mean size and polydispersity was evaluated. Initially, zein (45 or 60 mg) was dissolved in a hydroalcoholic solution (80 or 85%), and gallic acid (15 or 20 mg) was prepared either in water or in PEG aqueous solution (10–60 mg). The aqueous to organic solution ratio was 1:2 or 1:3 (*V*/*V*). Zein solution was added dropwise into the gallic acid aqueous solution (containing or not PEG) under moderate magnetic stirring at room temperature, and the particles were immediately formed. The dispersion was stirred for 120 min for ethanol evaporation. The unloaded gallic acid was separated from the nanoparticles by ultracentrifugation (25 249×*g*, at 25 °C for 20 min; model Z 36 HK; HERMLE Labortechnik GmbH, Wehingen, Germany) and the supernatant was stored for further analysis. For physicochemical characterization, nanoparticles were subsequently lyophilized. Unloaded nanoparticles were prepared by the same method but without the addition of gallic acid into the aqueous phase.

### Determination of mean particle size, size distribution, zeta potential and morphology

Mean particle size, polydispersity index (PDI) and size distribution were determined by dynamic light scattering (90Plus; Brookhaven Instruments Corp., Holtsville, NY, USA). Zeta potential was evaluated using a Zetasizer Nano S90 (Malvern Instruments, Malvern, UK). Morphology of the nanoparticles was investigated by scanning electron microscopy (SEM) (MIRA3 LM; Tescan Orsay Holding a.s., Brno, Czech Republic) at an accelerating voltage of 20.0 kV.

### Fourier transform infrared spectroscopy

Fourier transform infrared spectroscopy (FTIR) analysis was performed to monitor any structural changes related to the functional groups of nanoparticles and gallic acid due to nanoencapsulation. The FTIR spectra of the samples (gallic acid, zein, PEG and zein-PEG nanoparticles loaded with gallic acid) were recorded as transmittance in the scanning range of 400-4000 cm^-1^ (VERTEX 70; Bruker, Billerica, MA, USA).

### X-ray diffraction

X-ray diffraction (XRD) analysis of the samples was performed using an X-ray diffractometer (X-DS Phaser; Bruker, Karlsruhe, Germany). Thermograms were collected over an angular range from 2*θ*=10º to 60º in continuous mode using a step size of 2*θ*=0.02º and step time of 5 s.

### Encapsulation efficiency and drug loading

The encapsulation efficiency of gallic acid in zein nanoparticles was calculated indirectly. The supernatant containing non-encapsulated gallic acid obtained from the ultracentrifugation of nanoparticles was quantified by high-performance liquid chromatography (2695 alliance HPLC system; Waters Technologies, Inc., Milford, MA, USA). The mobile phase consisted of acetonitrile/water/0.5% acetic acid (54:28:18, *V*/*V*/*V*), and the flow rate was 0.9 mL/min in an isocratic elution. The photodiode array detector was set at 271 nm, and the total run time was 4 min. Encapsulation efficiency and drug loading, respectively, were determined as follows:



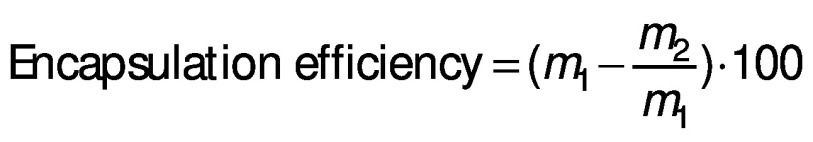





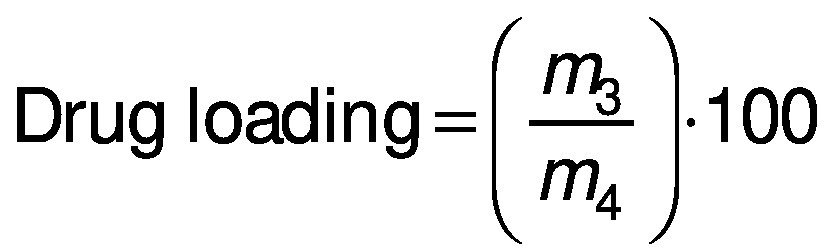



where *m*_1_ is the mass of gallic acid used for loading, *m*_2_ is the mass of gallic acid quantified in the supernatant, *m*_3_ is the mass of entrapped gallic acid and *m*_4_ is the mass of nanoparticles loaded with gallic acid.

### Stability in simulated gastric fluid and simulated intestinal fluid

Nanoparticles containing about 1.2 mg of gallic acid were dispersed in 5 mL of simulated gastric fluid (SGF; 50 mM KCl, 1% pepsin, pH=1.2). The samples were incubated at 37 °C under agitation at 150 rpm, and after 2 h the nanoparticle suspension was centrifuged at 25 249×*g* (model Z 36 HK; HERMLE Labortechnik GmbH) and introduced into simulated intestinal fluid (SIF; 50 mM KH_2_PO_4_, 15 mM NaOH, 1% pancreatin, pH=6.8) for 4 h. At specific time intervals (0.5, 1, 2, 3, 4, 5 and 6 h), samples were centrifuged (25 249×*g*, 25 °C and 15 min) and the removed volume was replaced with fresh medium to keep it constant. Finally, the supernatants collected at each time interval were filtered through a 0.22-μm filter and analyzed by HPLC to quantify the content of gallic acid released from the nanoparticles. The study was performed in triplicate, and the results were expressed as cumulative percentage of gallic acid released over time.

### Stability assay in food simulators

Two kinds of food simulants with 95 and 50% ethanol were used to perform the study. The 95% ethanol can be regarded as a simulant for fats, oil and fatty foods due to similar hydrophobicity, while 50% ethanol can be considered as a simulant for oil in water emulsions and alcoholic beverages (*24*).

Nanoparticles containing gallic acid (about 1.2 mg of the compound) were dispersed in 3 mL of the simulants. The samples were kept under stirring at room temperature and protected from light. At specific time intervals (0.5, 4, 8, 24, 48, 72, 96 and 120 h), samples were centrifuged  (25 249×*g*, 25 °C and 15 min) (model Z 36 HK; HERMLE Labortechnik GmbH) and the supernatants were collected and analyzed by HPLC (2695 alliance HPLC system; Waters Technologies, Inc.). After each sampling, the medium was replaced with a fresh medium to keep the volume constant. The study was performed in triplicate and the results were expressed as cumulative percentage of gallic acid released over time.

### Determination of antioxidant activity

The antioxidant activity was determined by the scavenging activity of 2,2-diphenyl-1-picrylhydrazyl (DPPH) radical, which is observed as a decrease in the absorbance at 517 nm, depending on the antioxidant concentration (*25*). Aliquots of different concentrations of gallic acid or gallic acid-loaded zein-PEG nanoparticles (5, 10, 20 or 40 μg/mL) were incubated in a shaker at 150 rpm and 37 °C. At predetermined times (0 and 24 h), aliquots of the samples were added to DPPH (25 μM) and filled with phosphate buffer (50 mM, pH=7.4) to the final volume of 200 μL. The absorbance was measured at 517 nm in a microplate reader (SpectraMax 190; Molecular Devices LLC, Sunnyvale, CA, USA). All determinations were performed in triplicate, and the percentage of DPPH inhibition was calculated as follows:



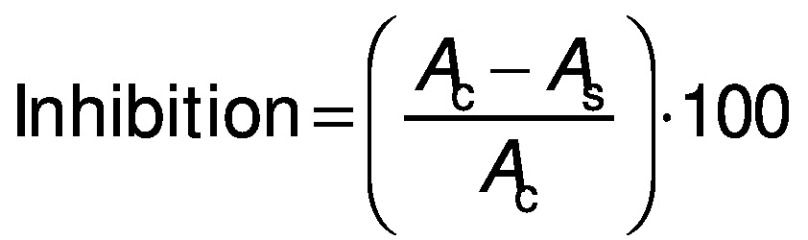



where *A*_c_ is the absorbance of the control and *A*_s_ is the absorbance of the sample.

### Statistical analysis

All data were reported as mean value±standard deviation (S.D.). Statistical analysis was conducted by one-way analysis of variance (ANOVA) followed by Tukey’s *post hoc* test for comparing groups using OriginPro v. 2018 SR1 b9.5.1.195 (*26*). Statistical significance was set at p<0.05.

## RESULTS AND DISCUSSION

### Preparation of nanoparticles and their characterization

The influence of some formulation factors such as polyethylene glycol (PEG), gallic acid and zein content, volume fraction of hydroalcoholic solution and aqueous to organic phase ratio on the mean size and polydispersity index (PDI) of zein nanoparticles containing gallic acid was evaluated, and results are shown in Table 1.

**Table 1:** Effect of some process variables on nanoparticle size (*d*) and polydispersity index (PDI)

Formulation	*m*(PEG)/mg	*m*(GA)/mg	*m*(zein)/mg	*φ*(hydroalcoholic solution)/%	*V*_organic solvent_/*V*_water_	*d*/nm	PDI
F1	0	20	60	80	1:2	−	−
F2	10	20	60	80	1:2	−	−
F3	20	20	60	80	1:2	−	−
F4	30	20	60	80	1:2	−	−
F5	40	20	60	80	1:2	−	−
F6	50	20	60	80	1:2	(366±35)^a^	(0.24±0.05)^a^
F7	60	20	60	80	1:2	(338±28)^ab^	(0.22±0.02)^a^
F8	60	15	60	80	1:2	(321±15)^ab^	(0.22±0.03)^a^
F9	60	15	45	80	1:2	(294±10)^b^	(0.21±0.01)^a^
F10	60	15	45	85	1:2	(287±15)^b^	(0.19±0.01)^a^
F11	60	15	45	85	1:3	(180±20)^c^	(0.18±0.01)^a^

The formation of nanoparticles was not observed; high agglomeration without redispersion. The results for *d* and PDI are expressed as mean value±S.D. (*N*=3). In each column, the same letters represent statistical equality and different letters statistical inequality (ANOVA and Tukey’s *post hoc* test) at p<0.05

It can be observed that without PEG, zein nanoparticles were not obtained (F1). A possible reason might be the aggregation of zein at neutral pH due to its isoelectric point around 6.2. It is well known that zein nanoparticles have poor colloidal stability, forming aggregates and precipitating in the formulation, losing their nanometric arrangement (*22*, *23*, *27*). Some strategies have been applied to improve the chemical and physical stability of zein nanoparticles, such as the inclusion of caseinate (*19*, *28*), chitosan (*27*), pectin (*29*), pluronic F127 and lecithin (*30*), sodium deoxycholate (*22*), lysine and caseinate (*31*), hyaluronic acid (*32*), and others. In this study, we evaluated the ability of PEG as a stabilizer to obtain zein nanoparticles with high colloidal stability. PEG was added in a range of 10 to 60 mg, although up to 40 mg, nanoparticles were not formed (F2–F5). When PEG was increased to 50–60 mg, nanoparticles were obtained. Possibly, a high PEG content contributed to better stabilization of zein nanoparticles, reducing the interaction between them through steric stabilization, thereby preventing their aggregation. Nanoparticles had a mean size of about 350 nm, without differences in the mean size between formulations F6 and F7. PEG is an anionic polymer, which has flexible hydrophilic chains, and it is widely recognized for its steric properties on nanoparticle surfaces, promoting the repulsion of particles (*33*-*35*). Zein as a weak polyelectrolyte forms complexes with other polymers by the driving forces of hydrophobic interactions and hydrogen bonds. These interactions improve the characteristics and stability of the resulting nanoparticles (*27*). After observing the formation of PEG-stabilized zein nanoparticles, we changed other parameters to reduce the mean size. When the amount of gallic acid was reduced (F8), a slight decrease in the mean size was observed. When the amount of zein was also reduced (F9), a significant decrease in nanoparticle size was observed. The increase in the volume fraction of hydroalcoholic solution did not affect the size (F10), compared to F9, but the increase in the volume fraction of aqueous phase (F11) significantly reduced mean size (<200 nm; p<0.05) of nanoparticles. F11 nanoparticle formulation was chosen as the ideal to continue the studies. According to Pascoli *et al*. (*36*), the main factors influencing zein nanoparticle size are the rate of injection of the organic phase into the aqueous phase, agitation speed and volume ratio.

**Fig. 1:**
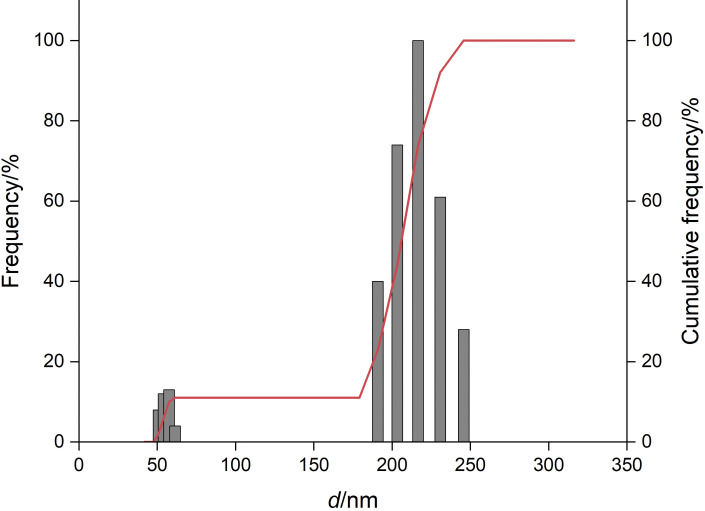
Size distribution of a representative sample of zein-PEG nanoparticles containing gallic acid

**Fig. 2:**
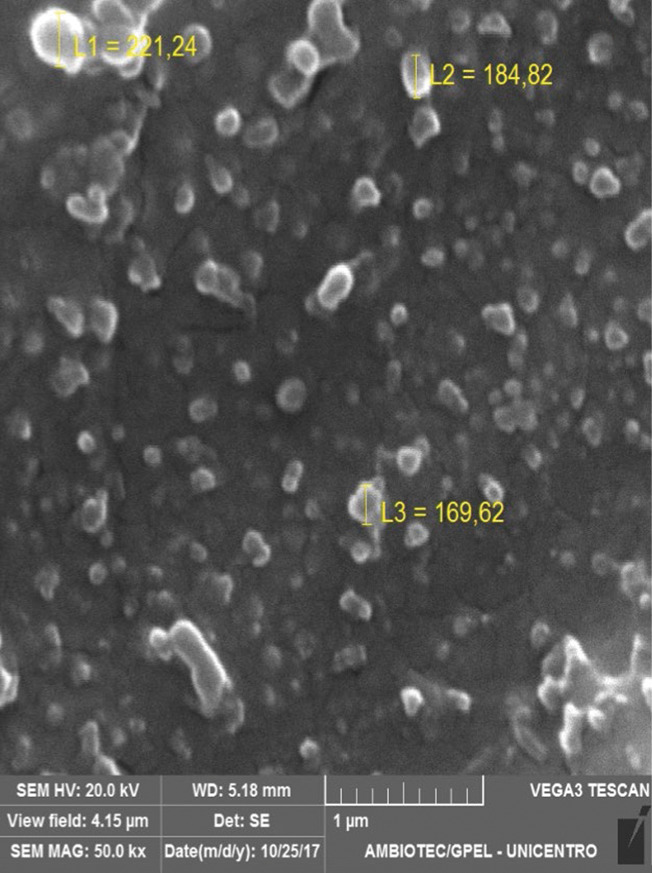
SEM image of the zein-PEG nanoparticles containing gallic acid

The nanoparticle size significantly affects their absorption and distribution in biological tissues; when intravenously administered, particles smaller than 20 nm are eliminated from the circulation within a few hours *via* the reticuloendothelial system, and particles larger than 300 nm are retained in the liver and spleen within minutes (*37*). Although the mean size is an important parameter for nanoparticle characterization, the size distribution is essential to predict their distribution. Narrow size distribution means a higher homogeneity. PDI is used to describe the degree of uniformity of a size distribution of particles. Values of 0.2 and below are considered acceptable in practice for polymer-based nanoparticle materials (*38*). The mean size of PEG-stabilized zein nanoparticles containing gallic acid was about 180 nm, and the size distribution revealed that most of the nanoparticles (90%) were distributed in the range of 180-250 nm (Fig. 1) and PDI 0.18. SEM image showed spherical or slightly oval nanoparticles (Fig. 2) and the indicated size of nanoparticles corroborated dynamic light scattering analysis. Our results are according to the literature. Zein nanoparticles with particles size between 130 and 170 nm were obtained using sodium caseinate as the stabilizer (*19*). Zein-carboxymethyl cellulose nanoparticles had *d*=196 nm (*20*). The application of low amounts of sodium deoxycholate as stabilizer for zein nanoparticles was efficient to produce 100-nm nanoparticles; however, higher concentrations of stabilizers significantly increased the particle size and the aggregation (*22*). Zein nanoparticles stabilized with chitosan were obtained in a range of 196 to 378 nm, depending on the chitosan concentration (*21*).

The zeta potential of zein-PEG nanoparticles was (-20.3±0.1) mV. The electrostatic repulsion among the particles depends on the zeta potential. The higher the value the stronger the repulsion, thus the nanoparticles become more physically stable. A value close to ±30 mV is important for good long-term stability since large repulsive forces tend to prevent aggregation due to occasional collisions of adjacent nanoparticles. In addition, the surface charge is an important parameter in determining whether nanoparticles clump together in blood flow, cling to or interact with membranes with the opposite charge (*39*).

The encapsulation efficiency of gallic acid in PEG-stabilized zein nanoparticles was (39±4) % and the loading was (5.2±0.8) %. The encapsulation efficiency value was consistent with gallic acid solubility of approx. 20 mg/mL in water, demonstrating its hydrophilic characteristics. It is known that the encapsulation of hydrophilic compounds is a challenge due to their rapid partition into the aqueous phase (*40*, *41*).

**Fig 3:**
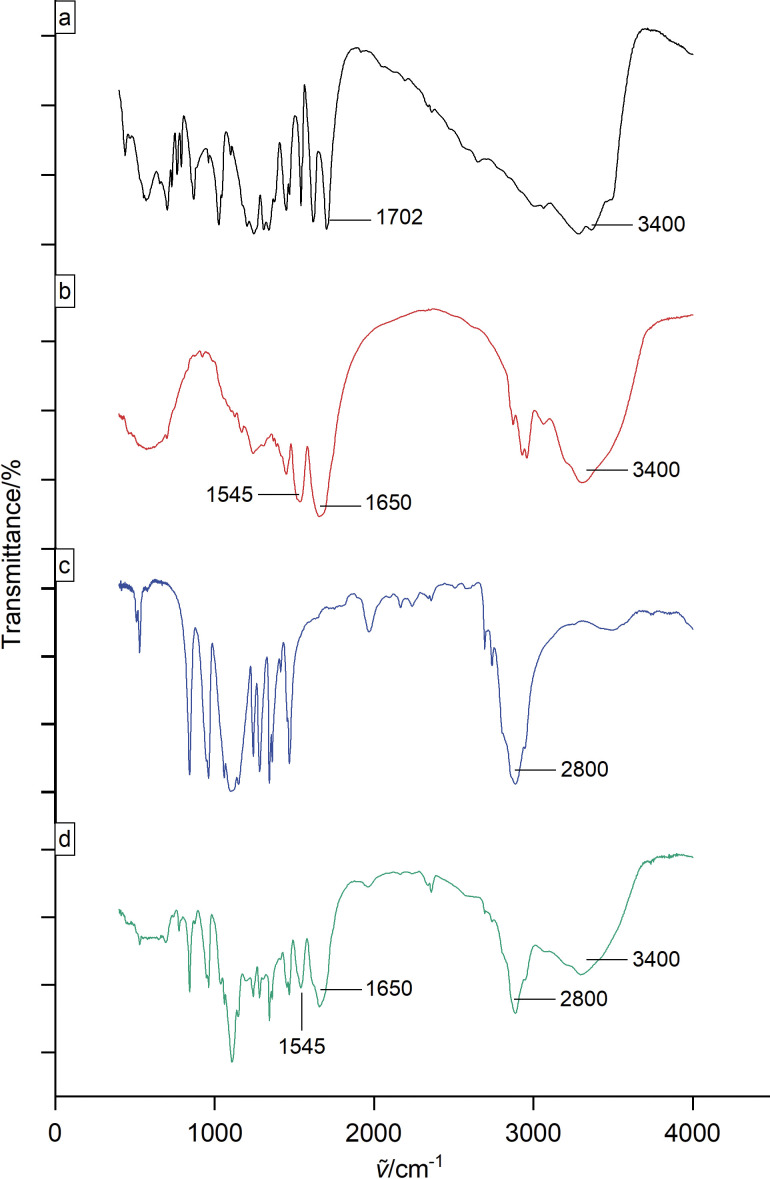
FTIR analysis of: a) gallic acid, b) zein, c) PEG and d) zein-PEG nanoparticles loaded with gallic acid

FTIR analysis served to identify changes caused or interactions induced by nanoencapsulation in the structure of gallic acid and zein. Gallic acid spectra (Fig. 3a) showed the characteristic bands at 3400 cm^-1^ due to O–H stretching and at 1702 cm^-1^ due to C=O stretching of carboxylic acid. The main vibrational features of zein FTIR spectra (Fig. 3b) include a broad peak at 3400 cm^−1^, which is due to N-H and OH stretching band, and two intense bands at 1650 and 1545 cm^−1^, corresponding to the amide bands, C=O vibrational stretching (amide I) and C-N and N-H stretching vibrations (amide II), respectively. PEG spectra (Fig. 3c) showed the main characteristic band at approx. 2800 cm^−1^ due to C-H stretching. Zein-PEG nanoparticles containing gallic acid (Fig. 3d) showed the same absorption peaks of the isolated compounds, except for the gallic acid band at 1702 cm^-1^, which was overlaid with the amide I band of zein. This means that there is no new bond formed or strong chemical interaction occurring due to the nanoencapsulation process.

**Fig. 4:**
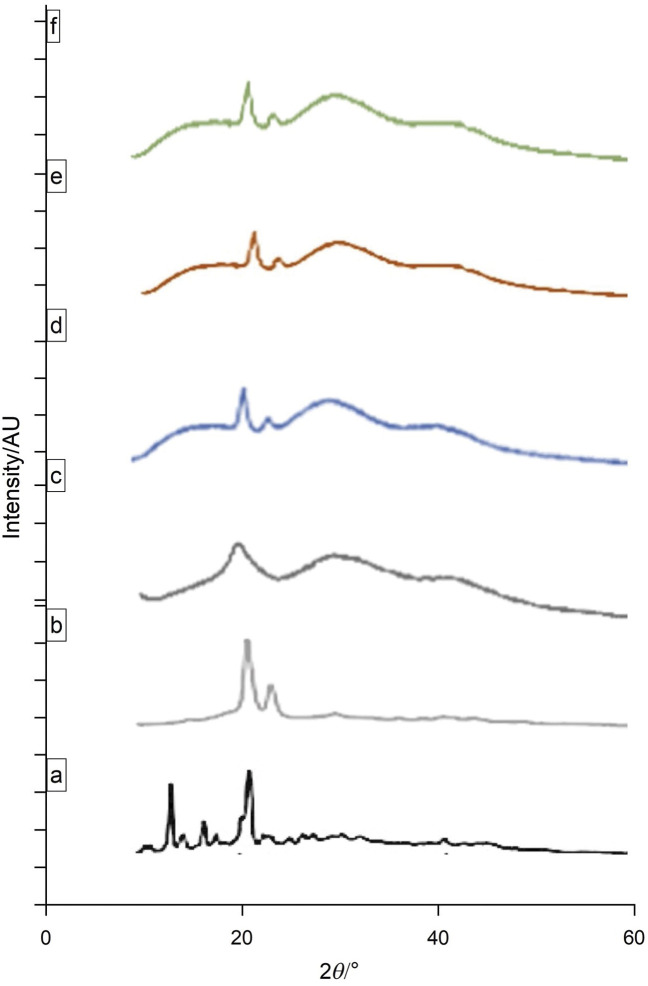
XRD analysis of: a) gallic acid, b) PEG, c) zein, d) physical mixture, e) unloaded nanoparticles and f) zein-PEG nanoparticles loaded with gallic acid

X-ray diffraction (XRD) analysis was carried out to verify the physical state of gallic acid after nanoencapsulation, and the results are presented in Fig. 4. XDR curve of gallic acid (Fig. 4a) showed a pattern of crystalline diffraction with well-defined peaks at 13.88°, 21.29° and 21.58°, according to the literature (*42*). XRD pattern of PEG (Fig. 4b) showed two intense diffraction peaks at 21.29° and 23.6°, characterizing its crystalline structure (*43*). XRD curve of zein (Fig. 4c) showed one diffraction peak at 20.25°, followed by two wide peaks, indicating a semi-crystalline structure. In the physical mixture (Fig. 4d), we observed only the peaks of PEG, with lower intensity, and the diffraction peaks of gallic acid did not appear, indicating a possible change in the orientation of gallic acid molecules with zein. The XRD curve of unloaded PEG-zein nanoparticles (Fig. 4e) was similar to the curve of gallic acid-loaded PEG-zein nanoparticles (Fig. 4f), indicating the loss of crystallinity of gallic acid after its nanoencapsulation. The absence of the diffraction peaks characteristic of the crystalline structure of the gallic acid is an indicative of a change in its crystallinity when incorporated into the nanoparticles, suggesting the amorphization of the compound. Amorphous form brings advantages for pharmaceutical compounds since this state improves drug solubility and bioavailability (*44*).

### Stability in simulated gastric and intestinal fluid

**Fig. 5:**
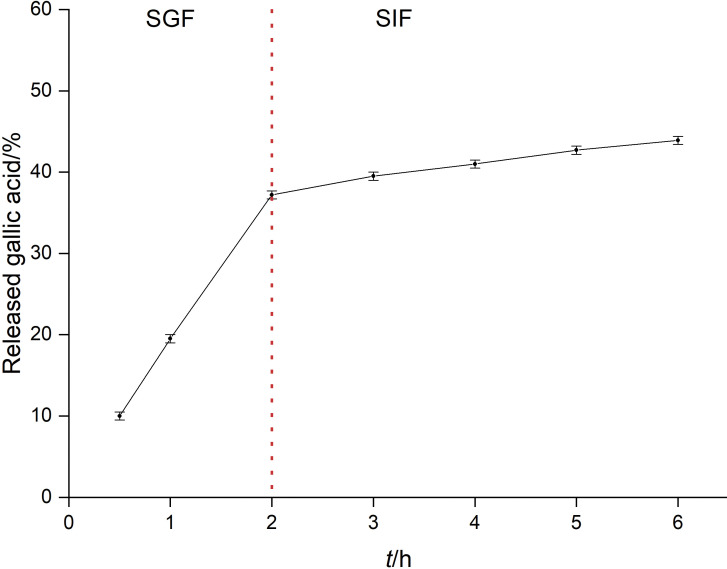
Gallic acid release profile from zein-PEG nanoparticles in simulated gastric fluid (SGF) (50 mM KCl, 1% pepsin, pH=1.2) and simulated intestinal fluid (SIF) (50 mM KH2PO4, 15 mM NaOH, 1% pancreatin, pH=6.8). The assay was conducted at 37 °C and 150 rpm for 2 h in SGF and for 4 h in SIF. Results were expressed as mean value±S.D. (*N*=3)

This assay was carried out to verify the ability of nanoparticles to protect gallic acid from its premature release in the gastrointestinal tract. Results are presented in Fig. 5. It can be verified that after 2 h of incubation of nanoparticles in SGF, about 37% of gallic acid was released. A possible enzymatic breakdown of zein may occur associated with the release of gallic acid molecules adsorbed on the nanoparticle surface (*45*). After 2-hour incubation in SGF, nanoparticles were incubated for 4 h in SIF, and the release of gallic acid was slower (about 7%), demonstrating higher stability of the nanoparticles in the SIF than in the SGF. Due to zein isoelectric point, at low pH the molecules have positive charge, which causes a rearrangement of the protein conformation interfering with the rate of drug release. In the alkaline medium, as the pH is closer to the isoelectric point of zein, the release rate of gallic acid is lower due to smaller change in zein conformation. The results show that PEG-stabilized zein nanoparticles can protect more than 50% gallic acid content under different pH conditions, indicating a potential oral formulation. The higher stability in SIF can have important implications since nanoparticles containing gallic acid can be absorbed through intestinal cells and reach the blood with an appreciable amount of drug loading for distribution to cells and tissues.

### Stability assay in food simulants

**Fig. 6:**
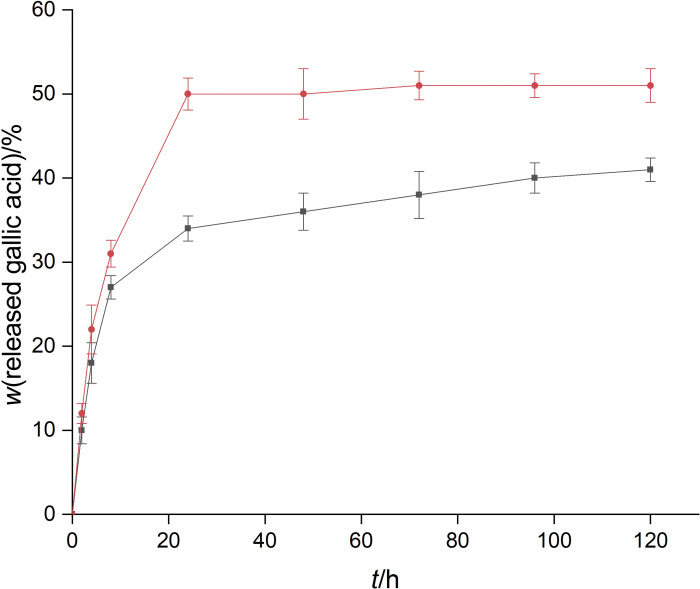
Gallic acid release profile from zein-PEG nanoparticles in 95% ethanol (red line) and 50% ethanol (black line)

Nanoparticles were introduced in food simulants to verify the gallic acid release profile. Two simulants were used, 95 and 50% ethanol. The first is a simulant of fats, oils and fatty foods because of their similar hydrophobicity. The 50% ethanol can be considered as a simulant of oil emulsions in water and alcoholic beverages (*13*). The gallic acid release in these media was evaluated over 5 days. The results are shown in Fig. 6. In both simulants, there was a burst release in 24 h, probably due to the desorption of gallic acid molecules from the nanoparticle surface. In 95% ethanol, about 54% gallic acid was released, while in 50% ethanol 35% gallic acid was released. After 24 h, a sustained release was observed until the fifth day. The gallic acid release was more sustained in 50% ethanol (p<0.05) since zein is more soluble in >70% ethanolic solutions. Liang *et al*. (*13*) obtained similar results with the release of epigallocatechin from zein-chitosan nanoparticles in these simulants. The results suggest that PEG-stabilized zein nanoparticles may be used to release gallic acid in fat and oily foods or alcoholic beverages, as nutraceutical supplementation or even as an antioxidant protector in foods.

### Determination of antioxidant activity

The results of DPPH inhibition at 0 and 24 h by unloaded or nanoencapsulated gallic acid are shown in Table 2. The antioxidant activity depended on the gallic acid concentration for both samples (p<0.05). The increase in gallic acid concentration resulted in higher radical inhibition (p<0.05) for both unloaded and gallic acid-loaded nanoparticles. However, time did not affect it (p>0.05). The highest DPPH inhibition of about 90% was achieved at higher concentration of gallic acid (unloaded or gallic acid-loaded nanoparticles) at 0 h and after 24 h. Blank nanoparticles did not show antioxidant activity. These results suggest that nanoencapsulation did not reduce the antioxidant potential of gallic acid.

**Table 2:** DPPH radical inhibition by gallic acid (unloaded or nanoencapsulated – formulation F11 in Table 1) in phosphate buffer (50 mM, pH=7.4) measured at *λ*=517 nm at 0 and 24 h

*γ*(gallic acid)/(µg/mL)	DPPH inhibition/%	
Unloaded gallic acid	Gallic acid-loaded zein-PEG nanoparticles
	*t*=0 h	
5	(25±3)^e^	(17±2)^f^
10	(49±3)^d^	(43±3)^d^
20	(81±3)^b^	(69±3)^c^
40	(92±1)^a^	(90±1)^a^
	*t*=24 h	
5	(21±3)^f^	(15±2)^g^
10	(47±2)^d^	(39±3)^e^
20	(79±2)^b^	(69±2)^c^
40	(91±1)^a^	(89±1)^a^

The results are expressed as mean value±S.D. (*N*=3).

The same letters in the same row represent statistical equality and different letters in the same row represent statistical inequality. Statistical analysis performed separately at 0 and 24 h (ANOVA and Tukey’s *post hoc* test) at p<0.05

DPPH is a stable free radical that is purple in a phosphate buffer solution (pH=7.4), with an absorbance band at 517 nm. In the presence of a scavenger molecule, a reduction of DPPH occurs, and it becomes colorless, and a decrease in the absorbance is observed. Gallic acid shows high scavenging activity against DPPH, due to the donation of hydrogen to the radical. Its activity is so strong that it can reduce six DPPH radicals per molecule, justifying the high inhibition observed in our results (*46*).

The importance of consuming antioxidants is due to oxidative stress causing deleterious effects on cells, aggravating cell aging, as well as contributing to the development of cardiovascular diseases, mutagenic or neurological alterations and the growth of cancerous tumors (*47*). Polyphenols are recognized by their antioxidant properties; however, the *in vitro* potential is not observed under *in vivo* conditions due to the pharmacokinetic drawbacks. Nanoencapsulation improves drug solubility, stability and bioavailability, which can expand various antioxidant-derived biological effects of polyphenols, such as gallic acid.

## CONCLUSIONS

Polyethylene glycol (PEG)-stabilized zein nanoparticles containing gallic acid were successfully produced by the liquid-liquid dispersion method. PEG was essential for the formation and stabilization of nanoparticles. With modification of some process variables, nanoparticles had reduced mean diameter, low polydispersity index, spherical or slightly oval morphology and negative zeta potential. Gallic acid was encapsulated in an amorphous state. PEG-stabilized zein nanoparticles were highly stable in simulated gastrointestinal conditions, and they can secure a release of a small amount of gallic acid in gastrointestinal tract before absorption. In food simulants, the gallic acid release was prolonged, suggesting the possibility of the application of nanoparticles in the food industry. Moreover, the process of nanoencapsulation did not reduce the antioxidant activity of gallic acid. The overall results suggest that PEG-stabilized zein nanoparticles are promising carriers for gallic acid delivery, and due to their antioxidant properties, they can be applied as adjuvants in diseases caused by oxidative stress and in the food industry as additives or for supplementation.
